# The London Hospital

**Published:** 1918-06-08

**Authors:** 


### THE LONDON HOSPITAL.

### Admission of Women Medical Students.

THE report to the Governors, presented to the Quarterly Court on June 5, states that the Lord Mayor's appeal on behalf of the Million Shilling Fund has resulted to date in a collection of £20,000.

The Lord Mayor has been appointed a Life Governor, and a tablet, with a suitable inscription, will be placed over a bed recording his Lordship's kind help.

Mr. Russell Howard, one of the hospital's surgeons, has kindly agreed to be in residence for the rest of the war. This is the most valuable assistance possible to the efficient running of the hospital, as the War Office has been forced to call up the entire junior surgical staff. In fact, had Mr. Russell not come forward in this manner to assist it is difficult to say how the work could have been carried on.

MEDICAL STAFF RESIGNATIONS.

Several resignations of members of the staff are reported. Dr. F. J. Smith has felt it necessary, partly for reasons of health, to leave the staff, and it is with great regret that the House Committee have accepted his resignation. Fortunately Dr. Smith has agreed to give voluntary help until the winter, when he proposes leaving London. Sir James Mackenzie, the well-known heart specialist, has also resigned. His service has been of the greatest possible value in the pursuit of cardiac research. Both these gentlemen have been appointed consulting physicians.

Mr. Jonathan Hutchinson also tendered his resignation, but has been persuaded to remain on duty for the duration of the war.

WOMEN STUDENTS.

An entirely new departure is to be taken by the Medical College in the month of October next, when, for the first time, women students will be admitted on equal terms with men. As a result of the good work which they have done, it is felt to be only right that women should be given educational opportunities here as they are elsewhere.

In consequence of the experience of the officials of the hospital during air-raids the House Committee has had reluctantly to decide on closing the basement as a shelter during a raid. Experience has shown that the work of the institution cannot be properly carried out with the basement full of the public.

EDITH CAVELL HOME.

The Home which has been built in memory of Edith Cavell, and which the President, Queen Alexandra, named the Edith Cavell Home rather than the Alexandra Home, has now been completed, and in the course of a short time the nurses will have taken possession of it. It is hoped that Her Majesty will graciously consent formally to unveil the tablet recording the fact that she named the Home the Edith Cavell Home, and that she will also unveil the bust which Sir George Frampton has so generously presented.

